# Radiographic techniques in screen‐film mammography

**DOI:** 10.1120/jacmp.v3i3.2572

**Published:** 2002-06-01

**Authors:** Thomas R. LaVoy, Walter Huda, Kent M. Ogden

**Affiliations:** ^1^ 4314 Kelsey Drive Syracuse New York 13215; ^2^ Department of Radiology SUNY Upstate Medical University 750 East Adams Street Syracuse New York 13210

**Keywords:** Mammography, Dose, MQSA

## Abstract

The objectives of this study were to document imaging physics parameters associated with mammography physics surveys, and investigate how the choice of tube potential affects average glandular dose (AGD) and x‐ray exposure time. Data from 60 mammography units were obtained pertaining to representative values of mAs, exposure time, half value layer, AGD and film density when acquiring phantom images. The survey of clinical systems showed that for a normal sized breast as represented by the mammography accreditation phantom, 60% of these units were operated at 25 kVp, and 33% at 26 kVp. Median exposure times were 1.14 s at 25 kVp and 0.73 s at 26 kVp. The median AGD was 1.62 mGy at 25 kVp and 1.51 mGy at 26 kVp. As expected, the choice of x‐ray tube potential did not significantly affect the median film density value of 1.5. Five clinical systems, all from different vendors, had measurements performed of the AGD and x‐ray exposure time as a function of x‐ray tube potential at a constant film density. For a typical clinical x‐ray unit, increasing the x‐ray tube potential from 25 to 28 kVp reduced the exposure time by 50%, and reduced the AGD by 26%.

PACS number(s): 87.57.–s, 87.59.Ek, 87.62.+n

## INTRODUCTION

There are currently about 10 000 mammography x‐ray machines in operation in the United States. Following the passage of the Mammography Quality Standards Act (MQSA) on October 27, 1992, each of these mammography units is required to undergo comprehensive physics tests that assess the average glandular dose and corresponding image quality.^1^ The American College of Radiology has developed an accreditation program that helps ensure that imaging equipment used to perform routine mammography is performing in a satisfactory manner. Methods for performing these physics tests have been standardized and are available in the scientific literature.^2^ ^,^ ^3^

It is of interest to document key imaging physics data that are routinely measured during physics surveys.^4^ ^–^ ^6^ Parameters that are of interest include the mAs, exposure time, half value layer, average glandular dose, and film density. Availability of these data will help physicists working in this area to compare their individual results with those normally expected, and assist in the identification of anomalous data. Regulators and inspectors will find this information helpful in the setting or adjusting of acceptable levels for key dose and x‐ray imaging parameters. In addition, documentation of physics information can provide a useful benchmark against which future trends can be compared.

The operation of a mammography unit requires an explicit choice of the x‐ray tube potential used to generate the image. Modification of the x‐ray tube potential has a major impact on several important imaging physics parameters including the AGD, exposure time, and x‐ray beam quality as determined by the half value layer (HVL). It is of obvious interest to investigate how changes in x‐ray tube potential on clinical mammography imaging systems affects these key imaging and dose parameters. A knowledge of the relative trade off between dose and image quality as a function of x‐ray tube potential will help the radiology community to optimize how this type of examination is performed.^7^

In this study, we document the results obtained on 60 mammography systems in the upstate New York area that are tested by a medical physicist in private practice (TRL). In addition experimental measurements were made on the most common types of mammography systems to investigate how the choice of x‐ray tube potential affects exposure time and average glandular dose.

## METHODS

### Survey practice

Data were acquired for 60 mammography imaging systems relating to the following: (a) choice of x‐ray tube potential for imaging a normal sized breast; (b) the corresponding values of mAs; (c) x‐ray beam HVL; (d) AGD; and (e) phantom film density for generating the routine ACR/MQSA phantom images. All units were located in central New York State, as far North as Alexandria Bay, as far South as Binghamton, as far West as Syracuse, and as far East as Utica. Data reported in this study was gathered from surveys made between June 1, 1998 and June 1, 1999. Table I lists the type of radiographic unit, together with the corresponding screen‐film combination used at the facilities that were included in this survey.

**Table I:** List of manufacturers and screen‐film combinations for the 60 units included in this survey.

Mammography Systems		Sterling	Kodak		Fuji			
Manufacture	Model	Microvision C	MIN‐R M	2000	E	AD	MA	Total
	M‐II	1		2				2
	M‐II E	1		2				3
LoRad	M‐III	5		9				14
	M‐IV	2		6		1		9
	TransPo		1		1			2
General Electric	800T						1	1
	DMR			4				4
Mammomat 2	1			4				1
Siemens	Mammomat	3		4		2	1	10
	3000							
Instrumentarium	Alpha IQ	3		2				3
	Alpha RT			2				2
Bennett	MF‐150	1				1		2
	Contour	1						1
Amerisys	Arisa M				2			2
Transworld	MAM‐CP	1		1				2
Philips	Diagnost UM				1			1
Total		19	2	30	4	3	2	60

## SURVEYS

All data were gathered during routine annual physics surveys, as currently required by the MQSA, on the mammography units. Measurements of half value layer, average glandular dose, and phantom density were made in accordance with ACR guidelines for performing these tests.^2^

When available, accreditation phantoms were exposed using each vendor's automatic mode of operation for selecting the kVp, target and filter combination. In all cases, the automatic mode selected a Mo target and Mo filter combination. If no automatic mode of operation was available, the kV setting indicated on the unit's technique chart was used. The kV and mAs value for the phantom exposure was recorded as displayed on the operator's readout for each unit. The time of exposure was also taken from the readout, when available. When no time readout was available, the displayed mAs value was divided by the nominal mA to obtain the exposure time. Errors in exposure time incurred by using a nominal mA were experimentally determined using a Keithley meter, and shown to be much less than 0.1 sec.

Every facility had a processor that had been checked by the standardized STEP test to measure relative processing speed. The STEP test uses a film and sensitometer brought in by the MQSA inspector to compare the facility processor to a reference FDA processor. Score must be within 20% of FDA processor to pass. All facilities complied with manufacturer recommendations for film‐screen combination, processor replenishment and temperature.

### X‐ray tube potential and imaging performance

Five units were chosen for detailed study as they represented the most common mammography units in use (see Table III). These units also had the most common range of features, such as auto kV and rhodium filter option. For each unit, accreditation phantom images were generated at four x‐ray tube potentials from 25 to 28 kVp in 1 kVp increments. During these experiments, the phantom density was kept constant at 1.50. There were a total of 20 phantom images (5 units× 4 measurements per unit) with an average film density of 1.50±0.05.

**Table III:** Variation of four mammography imaging parameters (mAs, time, HVL and AGD) with x‐ray tube potential (25 and 28 kVp).

Manufacturer	Model	mAs*	Time*	HVL*	AGD*
Siemens	M‐3000	107.5/50.6	0.72/0.38	0.315/0.349	1.42/1.09
GE	DMR	129/54	1.29/0.54	0.334/0.367	1.83/1.29
Instrumentarium	Alpha IQ	93/45	0.93/0.53	0.336/0.372	1.51/1.19
Lorad	M‐IV	119/53.4	1.2/0.53	0.291/0.322	1.60/1.18
Bennet	Contour	185.6/92.6	1.24/0.62	0.277/0.315	1.71/1.20

* First value is at 25 kVp/second value is at 28 kVp.

For each x‐ray tube potential, measurements were obtained of the mAs, exposure time, HVL and AGD. Relative values for each of these parameters were obtained as a function of x‐ray tube potential by normalizing the measured value relative to the corresponding measurements obtained at 25 kVp. The behavior of these four parameters as a function of x‐ray tube potential were obtained by taking an average of these relative values of the five clinical systems investigated.

## RESULTS AND DISCUSSION

### Survey results

For the 60 mammography units included in this study, 36 (60%) operated at 25 kVp and 20 (33%) operated at 26 kVp for the standard size patient (and ACR phantom). For the remaining four units, two operated at 24 kVp, one at 25.5 kVp, and one at 28 kVp. A detailed analysis was made of four parameters associated with the accreditation phantom image, including the mAs, exposure time, half value layer, average glandular dose, and resultant film density as a function of kVp. The resultant percentile values computed for these five parameters are summarized in Table II as for the units operated at both 25 kVp and at 26 kVp.

**Table II:** Statistical analysis of imaging performance characteristics found for mammography units operated at 25 and 26 kVp.

	mAs		Exposure time (s)		Half value layer (mm Al)		AGD (mGy)		Film density	
Percentile value	25 kVp	26 kVp	25 kVp	26 kVp	25 kVp	26 kVp	25 kVp	26 kVp	25 kVp	26 kVp
10%	80	72	0.84	0.51	0.293	0.310	1.39	1.26	1.39	1.42
30%	87	77	1.03	0.57	0.299	0.328	1.54	1.37	1.46	1.48
50%	95	84	1.14	0.73	0.309	0.331	1.62	1.51	1.51	1.52
70%	106	93	1.23	1.05	0.319	0.334	1.70	1.64	1.53	1.53
90%	122	98	1.39	1.24	0.327	0.341	1.81	1.75	1.55	1.56

The data relating to mAs show that the required value at 26 kVp is about 15% lower than that at 25 kVp. The range of mAs used to generate a phantom image is from ~70 to ~120 mAs. Since typical tube currents on mammography systems are about 100 mA, the resultant exposure times are expected to be of the order of 1 sec.

**Figure 1:**
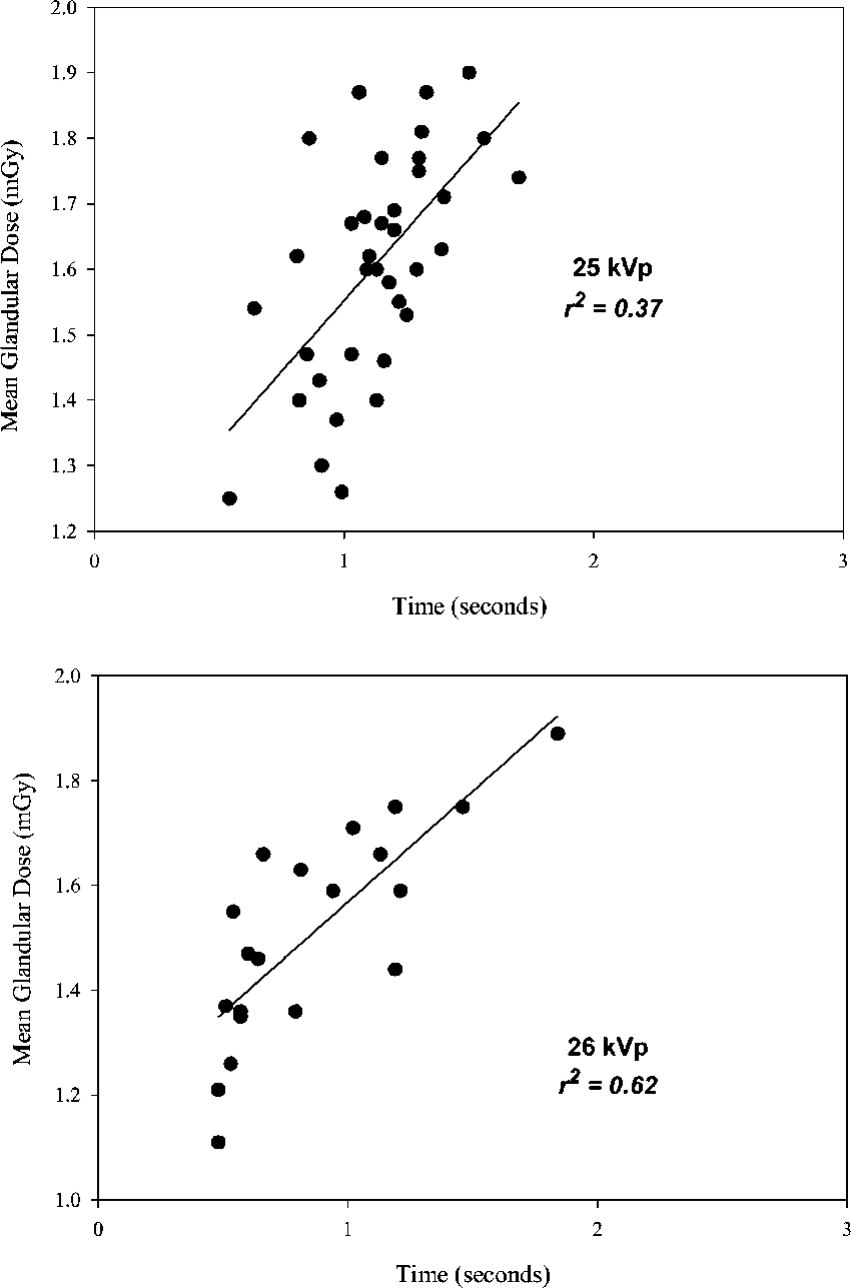
Average glandular dose versus measured exposure time for a breast with a thickness equivalent to the ACR accreditation phantom, and a 50% glandularity.

The median exposure time at 26 kVp (i.e., 0.73 sec) was about 36% lower than those associated with exposures performed at 25 kVp. Exposure time in mammography is important since a long exposure time would increase the probability of motion blur, and can also result in increased patient doses because of the effect of the film reciprocity law failure.^8^ ^,^ ^9^ It is of interest to note that at 26 kVp, the exposure times range from ~0.5 to ~1.3 sec and are markedly below the maximum exposure time of 2 sec recently recommended for optimal mammography.^7^ These data suggest that using an x‐ray tube potential of 25 kVp would offer an improved image contrast with little detriment to image quality (motion blur) or patient dose (film reciprocity law failure). Figure 1 shows the observed correlation between the average glandular dose and film exposure time at 25 kVp $\left(r^{2}=0.37\right)$ and 26 kVp $\left(r^{2}=0.62\right)$.

As expected, the half value layers at 26 kVp were about 7% higher than at 25 kVp. The HVLs given in Table II are markedly lower than the those values observed in the late 1980's (i.e., 0.38 mm Al), and comparable to those encountered by the late 1990's (i.e., 0.33 mm Al).^10^ The AGD data show that the dose penalty for using 25 kVp is approximately 7%, and that typical doses for this time period of 1.5–1.6 mGy are about a factor of 2 lower than the regulatory limit of 3 mGy. Typical AGDs in this study were 1.5–1.6 mGy, which is in excellent agreement to the values reported for the period 1995 through 1997 from MQSA survey results.^10^ These U.S. AGDs are somewhat lower than the 2.2 mGy per film reported for mammography screening in Australia.^11^

The data on film density in Table II show no differences between facilities who operate at 25 kVp and those using 26 kVp. The average film density was 1.5, and is at the lower end of the range of 1.5–2.0 that is currently deemed to achieve optimal film contrast.^7^ Higher film densities could be readily achieved by increasing the mAs value. Assuming a typical film gradient of about 3.2, an increase of film density from 1.5 to 1.8 would require an increase in exposure time of about 25% or so. Accordingly, film densities could be increased without having to increase the x‐ray tube potential, and without exceeding the two second exposure time recommended for optimal mammography.^7^

### X‐ray tube potential and imaging performance

Table III lists the values of four parameters (mAs, time, HVL, and AGD), obtained on five selected units, at x‐ray tube potential values of 25 and 28 kVp. The data in Table III reflect the absolute values of the four measured parameters as the x‐ray tube potential increases. It is notable that there are considerable differences in these parameters between the five investigated systems. At 25 kVp, for example, the mAs varies by a factor of 2, and the exposure times between the systems range from 0.7 to 1.3 sec. The resultant values of AGD, however, ranged from 1.4 to 1.8 mGy.

**Figure 2:**
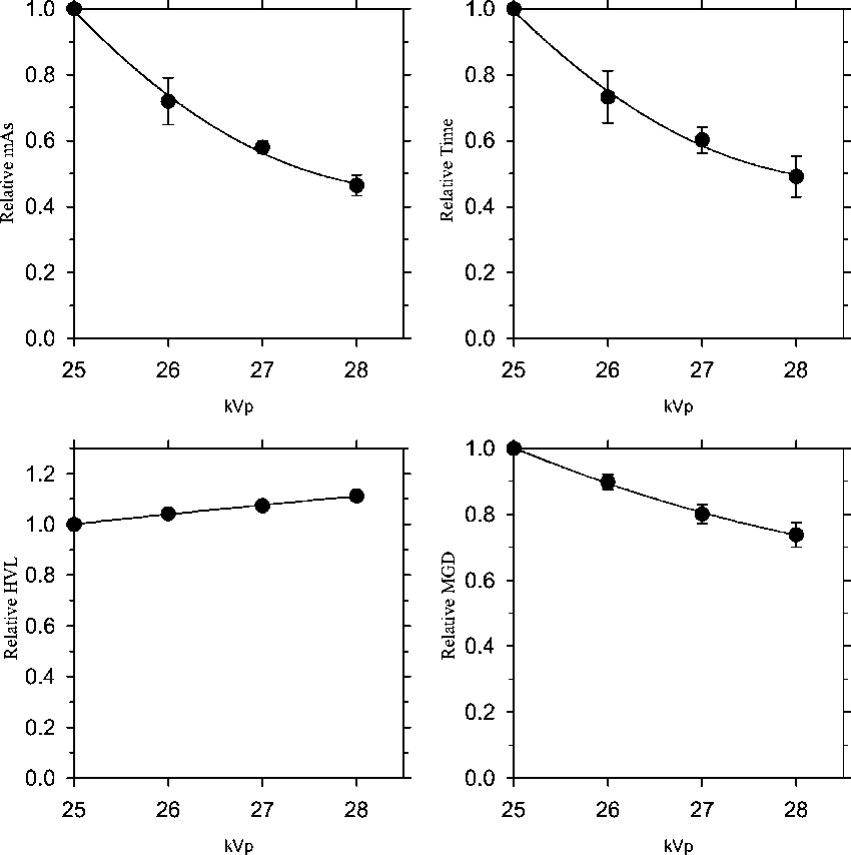
Relative change in radiographic parameters as a function of kVp: (a) mAs, (b) time, (c) half value layer, and (d) average glandular dose.

Figure 2 shows the relative values of four imaging parameters (mAs, time, HVL, and AGD) for five specified units (see Table III), where the data plotted represent mean and standard deviation (±σ) of the five clinical systems investigated. For each graph shown in Fig. 2, a least squares fit was obtained to the equation

$$\text{parameter}=\alpha \;\;\;{\text{kVp}}^{2}+\beta \;\;\;\text{kVp}+\gamma .$$

Table IV shows the values of the three fit parameters, as well as the corresponding coefficient of determination $\left(r^{2}\right)$.

**Table IV:** Summary of least square fit data for the equation (parameter $=\alpha kVp^{2}+\beta kVp+\gamma$ and the corresponding coefficient of determination $\left(r^{2}\right)$, given by the solid lines in Fig. 2.

Parameter	α	β	γ	$r^{2}$
mAs	0.04125	–2.361	34.24	0.996
time		0.03925	–2.246	32.61	0.995
HVL	$–1\times 10^{-3}$	0.0898	–0.6192	0.998
AGD	$9.75\times 10^{-3}$	–0.6050	10.03	0.998

Table V shows quantitatively how each parameter changes with kVp. Data shown in Table V provide definitive data on how changes in x‐ray tube potential will affect the relative values of mAs, exposure time, and corresponding AGD values. As such, these data will help to optimize mammography by quantifying those factors that modify dose and image quality as a function of x‐ray tube potential.^12^ For example, increasing the kVp from 25 to 26 kVp reduces the mAs and exposure times by about 25%, and reduces the AGD by about 10%. It is important to note that all the measurements presented in this study relate to a normal sized breast composed of 50% fibro‐glandular tissue and 50% adipose tissue, and additional studies are needed when imaging breasts of different thickness and composition.^13^

**Table V:** Relative parameter values as a function of kVp, normalized to 100% at 25 kVp. These data are obtained using the least square fit parameter given in Table III.

	Relative parameter value (normalized to 100% at 25 kVp)			
kVp	mAs	Time	HVL	AGD
25	99.4%	99.1%100%	100.1%
26	73.3%	74.6%	104.0%	89.2%
27	56.5%	58.7%	107.6%	80.7%
28	47.0%	49.7%	111.1%	73.7%
